# Endoscopic vacuum therapy in salvage and standalone treatment of gastric leaks after bariatric surgery

**DOI:** 10.1007/s00423-021-02365-9

**Published:** 2021-11-17

**Authors:** Ahrens Markus, Beckmann Jan Henrik, Reichert Benedikt, Hendricks Alexander, Becker Thomas, Schafmayer Clemens, Egberts Jan-Hendrik

**Affiliations:** 1 Department of Surgery, St. Vinzenz Hospital Dinslaken, Dinslaken, Germany; 2 Department of General, Thorax, Transplant and Paediatric Surgery, University Hospital Schleswig-Holstein, Campus Kiel, Kiel, Germany; 3 Department of General, Thorax, Vascular and Transplant Surgery, University Hospital Rostock, Rostock, Germany; 4 Israelite Hospital, Hamburg, Germany

**Keywords:** EVT, Leak, Complication of bariatric surgery, Endoscopic management, Bariatric surgery, Sleeve gastrectomy, Gastric bypass

## Abstract

**Introduction:**

Gastric leaks constitute some of the most severe complications after obesity surgery. Resulting peritonitis can lead to inflammatory changes of the stomach wall and might necessitate drainage. The inflammatory changes make gastric leak treatment difficult. A common endoscopic approach of using stents causes the problem of inadequate leak sealing and the need for an external drainage. Based on promising results using endoscopic vacuum therapy (EVT) for esophageal leaks, we implemented this concept for gastric leak treatment after bariatric surgery (Ahrens et al., *Endoscopy* 42(9):693–698, 2010; Schniewind et al., *Surg Endosc* 27(10):3883–3890, 2013).

**Methods:**

We retrospectively analyzed data of 31 gastric leaks after bariatric surgery. For leak therapy management, we used revisional laparoscopy with suturing and drainage. EVT was added for persistent leaks in sixteen cases and was used in four cases as standalone therapy.

**Results:**

Twenty-one gastric leaks occurred in 521 sleeve gastrectomies (leakage rate 4.0%), 9 in 441 Roux-en-Y gastric bypasses (leakage rate 2.3%), and 1 in 12 mini-bypasses. Eleven of these gastric leaks were detected within 2 days after bariatric surgery and successfully treated by revision surgery. Sixteen gastric leaks, re-operated later than 2 days, remained after revision surgery, and EVT was added. Without revision surgery, we performed EVT as standalone therapy in 4 patients with late gastric leaks. The EVT healing rate was 90% (18 of 20). In 2 patients with a late gastric leak in sleeve gastrectomy, neither revisional surgery, EVT, nor stent therapy was successful. EVT patients showed no complications related to EVT during follow-up.

**Conclusion:**

EVT is highly beneficial in cases of gastric leaks in obesity surgery where local peritonitis is present. Revisional surgery was unsuccessful later than 2 days after primary surgery (16 of 16 cases). EVT shows a similar healing rate to stent therapy (80–100%) but a shorter duration of treatment. The advantages of EVT are endoscopic access, internal drainage, rapid granulation, and direct therapy control. In compartmentalized gastric leaks, EVT was successful as a standalone therapy without external drainage.

**Supplementary Information:**

The online version contains supplementary material available at 10.1007/s00423-021-02365-9.

## Introduction

Gastric leaks (GL) constitute some of the most severe complications in bariatric surgery. The highest incidence of GL occurs at the proximal staple line in sleeve gastrectomies (SG) and at the gastro-jejunostomy in Roux-en-Y gastric bypasses (RYGB) [1–5]. Oftentimes, GL are diagnosed with a delay, mainly due to deferred occurrence of symptoms caused by the adjacent visceral fat, despite usage of drainage tubes. This delay complicates GL treatment. Therapeutic interventions for GL in bariatric surgery are therefore often delayed as well (> 2 days postoperatively). Such a delay is often sufficient for local peritonitis to manifest itself, making GL therapy even more challenging.

Over the past decade, the most common GL treatment has been early revision surgery and/or endoscopic stenting, with an increasing use of stent therapy in the last 5 years. Studies on employing endoscopic stenting for GL treatment in bariatric surgery report healing rates of 80–100%. However, such approaches come with risk of stent migration, insufficient leak sealing, persistent peritonitis, and dysphagia [6–14].

Based on our promising results of endoscopic vacuum therapy (EVT) in esophageal leakage after esophagectomy, we applied EVT management to GL after bariatric surgery [15, 16]. The advantages of EVT include endoscopic access and therapy control, granulation induction, and the permanent active drainage of inflammatory fluids. In addition, surrounding peritonitis and systemic infection are also reduced. Since 2011, we have routinely used EVT in our therapy management for GL in obesity surgery. In this retrospective study, we report on the treatment of 31 GL. Eleve of the 31 patients with GL were detected within 2 days and healed after a single revision surgery with suturing. In total, 20 of the 31 GL patients who became evident later than 2 days after primary surgery were treated with EVT. Other studies have described EVT in GL treatment as well; however, their observational datasets included a substantially smaller number of patients [13, 17–21].

## Materials and methods

In this single-center retrospective study, we analyzed 31 GL that occurred after 1006 bariatric operations between 2011 and 2017. The most prevalent procedures were sleeve gastrectomies (*n* = 521), proximal gastric bypasses (RYGB, *n* = 441), and mini-gastric bypasses (MGB, *n* = 12) (Table 1). Further 32 procedures constituted other bariatric operations such as gastric banding excision (*n* = 11), biliopancreatic diversion (BPD + DS, *n* = 2), SADI-Operations (*n* = 5), and distal gastric bypasses (*n* = 14). The mean BMI of our patient cohort was 50 (min = 36, max = 76) kg/m^2^. All 31 GL happened after primary sleeve gastrectomies and gastric bypasses (1 after a MGB). The contribution of comorbidities was comparable in the two subsets of SG and RYGB patients. SG was performed with a 42 Charrier bougie and routine additional sero-muscular suturing of the proximal staple line. For RYGB and mini-gastric bypass (MGB), we performed gastro-jejunostomy as a side-to-side linear stapler anastomosis. All obesity operations were performed laparoscopically without conversions. In all cases, we used the Echelon Flex™ Stapler (Ethicon, Johnson&Johnson), with green and golden magazines without buttressing. The routine hospital stay in non-complicated cases was 5 days.

**Table 1:** Overview of gastric leaks in the Roux-en-Y gastric bypass (RYGB), sleeve gastrectomy (SG), and mini-gastric bypass (MGB) groups

Gastric leaks	*n*	SG (*n* = 521)	RYGB (*n* = 441)	MGB (*n* = 12)
Early leaks (≤ 7 days postoperative), *n*	22	15	7	0
Late leaks (> 7 days postoperative), *n*	9	6	2	1
Total leak number, *n*	31	21	9	1
Proportion of leaks per procedure, %		4.0	2.0	

### Leaks, classification, and management

The GL were classified by the time of diagnosis: early GL ≤ 7 days postoperatively, and late GL ≥ 8 days postoperatively. This classification is inspiered by Weiner et al. and Csendes et al. who used a third intermediary group after 5–7 days [22, 23]. For first signs of early GL, we analyzed CRP > 100 mg/l. Late GL had pains in the upper abdomen and fever as first signs.

In all cases of early GL and 5 cases of late GL, we proceeded as follows: (1) re-laparoscopy, drainage, and overstitching of the GL; (2) in persistent GL, endoscopy and EVT. All GL patients received antibiotics and parenteral or nasal tube feeding. As we gained experience from previous procedures, we performed primary EVT without surgical revision on 4 patients who had a late GL with good compartmentalization (depicted by CT scan). Iv antibiotics were given from the day of GL diagnosis for 3–5 days depending on blood values. When GL sealing was achieved via the intracavital positioned endosponge, non-solid enteral feeding was possible. In cases of inadequate sealing, a naso-enteral tube was inserted and enteral tube feeding was performed. Whenever enteral feeding was impossible, we performed parenteral feeding.

### Endoscopic vacuum therapy technique

**Fig. 1:**
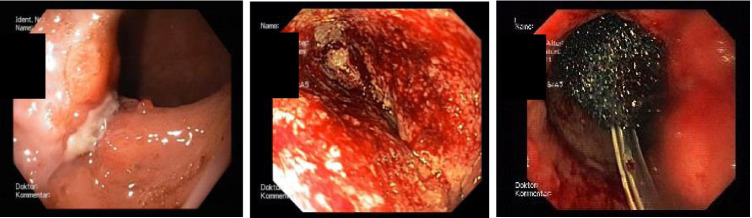
Use of EVT in an example of GL in a sleeve gastrectomy staple line in the run of time. The left picture shows a gastric leak (GL) in the proximal staple line after sleeve gastrectomy. The picture in the middle shows the view through the GL into the cavity with good endoscopic vacuum therapy (EVT)-induced granulation. The right picture shows an EVT sponge in the decreased hole and progressing granulation

We used the Endo-Sponge System (B. Braun, Melsungen, Germany). A polyurethane sponge with a fixed drainage was tailored to the size of the extraluminal cavity. We positioned the endosponge intracavitary in all EVT cases and applied a permanent suction of 75 mmHg. For greater leaks, we applied up to 120 mmHg. In cases of great cavities, we applied 2 endosponges intracavitary (*n* = 2). In cases of small leaks, we dilatated the leak so that an intracavitary positioning of a sized endosponge was possible. Changes of the endosponge were carried out every 2 days within the first week to allow for a tight initial therapy control. In subsequent weeks, endosponge changes were carried out every 3 days, since endosponge changes in later stages can cause sponge adhesions and leak injuries, based on our experience. We reduced the size of the sponge comparing to the increasing granulation of the leak and the cavity. Following the conventions in the GL treatment literature, we defined GL as healed when the wound cavity was smaller than about 1 cm in radius and 2 cm in depth [15, 16]. EVT was then terminated (Fig. 1).

We routinely performed the EVT Implantation or changing in Propofol© sedation and continuous ECG and oxygen monitoring in all patients. An intubation anesthesia for an EVT change was not necessary. During and after the endosponge changes, an intensive care stay was not necessary.

### Statistical analysis

The data was analyzed using SPSS for Macintosh, version 21.0 (IBM Corporation, New York, USA). All distributions and frequencies of medical data were compared by Fisher ‘s exact test.

### Ethical approval statement

Ethical approval for this study was granted by the University Hospital Schleswig–Holstein, Campus Kiel in 2013 (processing no. D427/13).

## Results

### Distribution of leaks per bariatric procedure

The overall leakage rate was 3% (31 of 1006 operations): 21 in 521 SG (4%), 9 in 441 RYGB (2%), and 1 in 12 MGB. Further 32 procedures—constituting other bariatric operations such as gastric banding excision (*n* = 11), biliopancreatic diversion (BPD + DS, *n* = 2), SADI-Operations (*n* = 5), and distal gastric bypasses (*n* = 14)—happened without any gatric leaks. The rate of late GL was more than twice as high after SG, compared to gastric bypasses (21 vs. 10, Table 1). The 22 early GL occurred during hospital stay and the 9 late GL occurred after admission. The 22 early leaks showed an increase in CRP (> 100 mg/dl) as a first sign of a potential GL. Nine late leaks occurred after admission and manifested themselves through fever or pain in the upper abdomen as first symptoms and CRP > 100 mg/dl in the further diagnostics.

All GL occurred after primary operations. In contrast to data from literature, we have not observed any GL after bariatric re-operations in our study.

### Therapy course overview

**Fig. 2:**
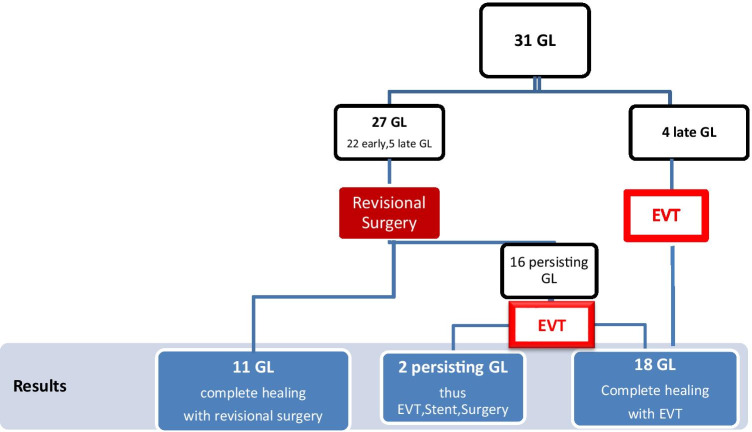
Overview of the therapy course in 31 patients with a gastric leak (GL)

In 27 of the 31 patients suffering postoperative GL, the first step was re-laparoscopy and GL suturing (Fig. 2). When GL re-operation took place within 2 days of bariatric surgery (*n* = 11), suturing led to complete healing. When re-operation took place after 2 or more days, suturing was insufficient and we added EVT. As we gained experience from previous procedures, we performed EVT as standalone therapy without revisional surgery or external drainage in 4 late GL cases. These 4 GL occurred more than 4 weeks after surgery. CT scans showed good compartmentalization of the GL and the EVT as standalone therapy led to healing in all 4 cases. In 2 late GL, neither surgery, nor extended EVT, nor changing to stent therapy led to healing.

During the data analysis, we divided into 2 therapy groups. The first group is the surgery group in which revisional surgery was conducted within 2 days after bariatric surgery sufficiently. The second group represents the EVT group in which revisional surgery was not sufficient, and hence, EVT was either added or performed as standalone therapy.

### Surgery group: revisional surgery within 2 days: *n* = 11

Eleven early GL could be re-operated within 2 days after surgery and were cured by revisional surgery, suturing, and percutaneous drainage (Fig. 2). At re-laparoscopy, we found a modest degree of local peritonitis. In 4 of these cases, a conversion to open surgery was necessary; two cases were RYGB, in which a new gastro-jejunostomy was performed (Table 2).

**Table 2:** Overview of the surgery group, *n* = 11: gastric leaks (early GL) detected and re-operated within 2 days after primary surgery and complete healing by re-laparoscopy and suturing

Surgery group	*n*	Suture	New gastro-jejunostomy	Conversion to open surgery
Early GL after SG, *n*	8	8	0	2
Early GL after RYGB, *n*	3	1	2	2
Tota GL, *n*	11	9	2	4

### EVT group: insufficient revisional surgery later than 2 days and added EVT or EVT as standalone: n = 20

In 16 patients where the GL were detected later than 2 days after bariatric surgery, revision surgery was not sufficient and EVT was performed (Fig. 2). The average duration of EVT was 17 days (Table 3). In patients with late GL (*n* = 9), the mean duration of EVT was shorter (15 days). Initially, we performed surgical revision with every patient diagnosed with a late GL. As we gained experience from previous procedures, standalone EVT therapy (without surgical revision) was performed in four patients with late GL leading to complete healing within 12 days.

**Table 3:** Overview of the EVT therapy, *n* = 20: mean time and endosponge changes

Leakage	Mean EVT time (days)		Mean number of endosponge changes	
	After rev. surgery	EVT standalone	After rev. surgery	EVT standalone
Early GL (*n* = 11)	19 days		7	
Late GL (*n* = 9)	15 days	12 days	6	5
Total GL (*n* = 20)	*n* = 16	*n* = 4	*n* = 16	*n* = 4
Mean total	17 days		6	

**Fig. 3:**
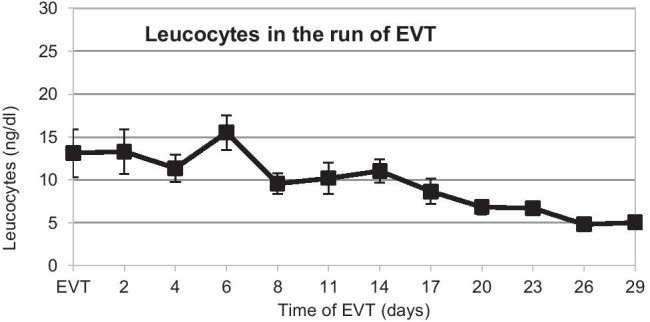
Course of leucocytes in 20 patients treated with endoscopic vacuum therapy (EVT)

**Fig. 4:**
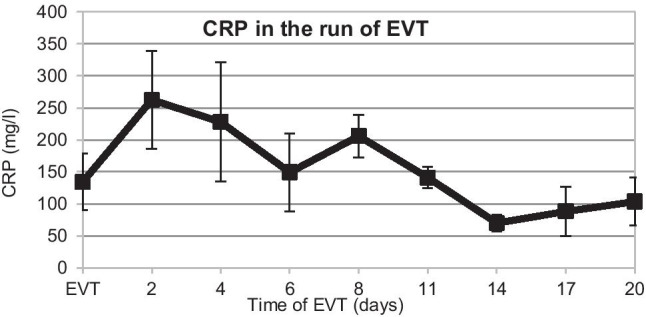
Course of C-reactive protein (CRP) in 20 patients treated with endoscopic vacuum therapy (EVT)

In summary, EVT led to complete healing in 18 of 20 GL (EVT healing rate 90%). Septic infection and local peritonitis were regulated and controlled using EVT within 2 days (Figs. 3 and 4). In 2 cases of late GL after SG, leaks were not sufficiently cured by either re-laparoscopy, EVT, or conversion to stent therapy. In one of these cases, even an anastomosis of the GL with a Roux-en-Y limb was insufficient, so ultimately a complete gastrectomy was necessary. The other case was a 68-year-old patient suffering from chronic hypertensive cardiomyopathy who passed away at an ischemic cardiac arrest during a planned endoscopic endosponge changing, despite immediate maximum intensive care and previous infection control. It was the fifth EVT change at this patient and we performed the endoscopy routinely in Propofol© sedation under continuous monitoring.

### Characteristics of patients with GL (surgery group vs. EVT group)

The EVT group showed a higher incidence of comorbidities in comparison to the surgery group (Table 4). In the EVT group, CRP (the first sign of early GL) increased after more than 2 days. Both the BMI and age of patients with GL were evenly distributed.

**Table 4:** Distribution of BMI, comorbidities, age, and first evidence of gastric leak (C-reactive protein > 100 mg/l) in the surgery and endovascular vacuum therapy (EVT) groups (*n* = 31)

	Surgery group	EVT group
*n*	11	20
BMI, mean (range) (kg/m^2^)	51 (76–41)	50 (64–41)
Age, mean (range) (years)	49 (52–27)	39 (58–26)
Diabetes mellitus, *n*	3	6
SAS/COPD, *n*	4	7
GERD, *n*	2	5
Detection of CRP > 100 mg/l after bariatric operation (days)	1–2	> 2

### Intensive care and feeding

After revisional surgery, patients stayed for 1 day in an intensive care unit. Longer or further intensive care stays were not necessary. Intravenous antibiotics were administered for 3–5 days. In all cases, the inflammatory blood parameters decreased within this timeframe, so that no patient required longer antibiotic therapy.

In one-third of all cases, a complete sealing of the leak was achieved through the initial intracavitary endosponge already, and liquid enteral feeding was performed. In another third, the GL sealing was incomplete, and hence, an endoscopic naso-enteral feeding tube had been inserted and enteral tube feeding was possible. For the last third, we could neither complete GL sealing via intracavitary endosponge nor successfully place a naso-enteral tube via an endoscope. Thus, parenteral feeding was performed. In the second week of EVT, 80% of the patients could be enterally fed with liquid nutrition when the extraluminal cavity and the GL size decreased under EVT.

### One-year follow-up after EVT

**Fig. 5:**
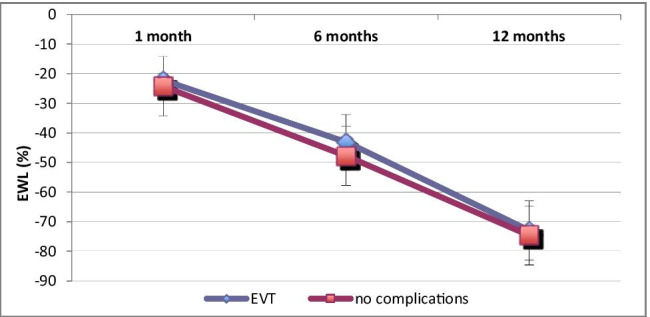
Excess weight loss (EWL) after obesity surgery. Patients with EVT-treated gastric leak (*n* = 19, 1 patient died) vs. patients with no complications (*n* = 480)

In the 1-year follow-up, there were no differences in loss of weight and comorbidity reduction between patients undergoing uncomplicated bariatric surgery and patients with an EVT-treated GL (Fig. 5). There were no EVT-related complications such as dysphagia or chronic gastric fistulas.

## Discussion

The fundamental problem about sufficiently managing GL after bariatric surgery is the delayed onset of symptoms, primarily caused by excess visceral fat and thus initial occult peritonitis. For GL detection, the diagnostic value of blood tests for inflammatory parameters or radiological diagnostics is limited. In particular, SG suffers from the dilemma of high intra-pressure in the gastric sleeve [1–3]. Drastic reduction of stomach volume and the matter of an intact persisting pylorus leads to high pressure in the gastric tube and a higher rate of GL at the proximal staple line in the fundus. This is mainly due to a slim fundus wall (only two layers of muscular lining). In studies, there was no proof of an ischemic cause for GL after sleeve gastrectomies [2, 3, 5, 24]. At the gastro-jejunal anastomosis of gastric bypasses, an ischemic cause of an anastomosis leak is known. The rate of GL was reported to be 2–4% in SG and 1–2.3% in RYGB [4, 5, 23, 25–27].

In the current literature, endoscopic stent implantation is increasingly used for GL treatment, with a treatment duration between 21 and 240 days [1, 6–10]. The rate of full recovery was reported to be 75–100%. Another endoscopic approach is clip techniques like “over the scope clip” (OTSC). Several studies in the literature reported healing rates of 60–70% for gastro-intestinal leaks [1, 12, 23]. However, these studies included both bowel perforation and leak sealing directly after full wall excision in the GI—thus, they did not exclusively focus on gastric leaks. A current study from 2021 on GL after sleeve gastrectomy using OTSC techniques showed only low efficacy [28]. A further endocopic approach is the endoscopic suture technique. Only case reports exist about the use of this technique for GL treatment after bariatric surgery. In a detailed review, Ge and Thompson (2020) summarized that endoscopic suturing is advantageous for acute perforation management, whilst being suboptimal for long-term management of non-acute fistulas [29]. Further approaches included CT-supported percutaneous drainage. However, its shortfalls are long treatment times and a failure rate of more than 50%. Surgical alternatives for SG leaks are a change to RYGB, a leak anastomosis with a Roux-en-Y limb, or a complete gastrectomy [5, 7, 25].

In accordance with current literature findings, up until 2015, we performed early revision laparoscopy with GL suture and drainage as our primary/default approach. In our cohort of 31 patients, 11 early GL showed increased CRP levels beyond 100 mg/l within 2 days of surgery and were admitted to re-operation on the same day. Local peritonitis was low. In all these 11 cases, surgical revision led to a beneficial outcome and no further treatment was needed (Table 2). In 16 cases of patients with GL detection later than 2 days, revision surgery failed due to advanced peritonitis and we performed EVT (Fig. 2). As we learned from previous procedures, we performed EVT alone without revisional surgery in 4 late leaks that occurred later than 4 weeks. CT scans showed good compartmentalization.

The overall healing rate with EVT was 90% (18 out of 20 GL). The mean treatment time of EVT was 17 days (Table 3). For the 4 cases with late leaks where standalone EVT was applied, the mean EVT timespan was 12 days. These results clearly show the advantages of EVT for patients with GL after bariatric surgery. More rapid wound granulation and permanent internal active suction drainage, especially in local peritonitis, are unambiguously a proficient approach in interventional treatment of GL. For patients in the EVT group, peritonitis and incipient sepsis were controlled within 2 days (Figs. 3 and 4). EVT and the nasal feeding tube were well tolerated, and patients were mobilized quickly. Directly after EVT as well as 3, 6, and 12 months after bariatric surgery, there were no complications such as dysphagia, esophagitis, malnutrition, or upper abdominal complaints. Patients’ weight loss was comparable to that of patients without postoperative gastric leakage (Fig. 5). Only a few papers report about GL treatment using EVT after obesity surgery. Furthermore, their sample size constitutes only up to 6 cases [13, 17–21]. Leeds et al. [21] reported from 8 GL and Archid et al. [30] from 9 GL a similar healing rate of 90% when EVT was used for GL treatment after SG.

Our data shows that suturing after the second day post bariatric surgery is not sufficient (16 of 16 cases). We conclude that after 2 days, EVT or stent therapy should be performed instead of suturing techniques. In EVT, we think it is crucial to place the endosponge intracavitary to drain and reduce the extraluminal GL cavity. Once a GL sealing could be reached by intracavitary endosponge positioning, liquid enteral feeding is possible.

In two cases of late leaks after SG, EVT was unsuccessful. Neither EVT, conversion to stent therapy, nor an anastomosis of the leak with a Roux-en-Y limb led to a positive outcome. In one of these cases, a gastrectomy was necessary. The other patient with a high-grade cardiomyopathy died during therapy endoscopic stent changing of ischemic cardiac arrest, despite maximal intensive care and controlled infection. Adequate granulation of the leakage region and its surrounding tissue could not be achieved in these patients. This might constitute a limitation of EVT and stent therapy.

In recent years, the literature has frequently reported the usage of stent therapy for treatment of GL as either stand-alone therapy or after failed revision surgery [6–12, 23, 31]. Comparison of our EVT data with data from patient cohorts after stent therapy showed the same cure rate (EVT 90% vs. stent 75–100%). The advantages of stent therapy are the one-time use and the possibility of normal enteral feeding. Two to three endoscopies are regularly needed for stent therapy. The most common disadvantages are stent migration (30-50%) and the necessity of external drainage of the wound cavity which is often challenging in obesity patients [1, 6–10, 13]. Kanters et al. [14] reported in an MBSAQIP database analysis of 275 stent therapy in GL after bariatric surgery that 50% patients with GL need more than one stent and a higher likelihood of readmission.

The main disadvantages of EVT are the need for repeated endoscopic interventions (every 2–3 days) and feeding via a tube or parenterally. On the other hand, EVT setting offers specific advantages such as permanent internal drainage, faster GL granulation, and endoscopic on-site therapy control [18–20].

Stent therapy has a cost advantage over EVT, since the latter requires a higher number of needed endoscopies and endosponge changes. Meanwhile, material costs are comparable (1 stent: ca. €700–1000 vs. 6 endosponges: €900–1000^1^). For EVT, the costs of materials are added to the costs for parenteral or tube feeding (€7/day, in average €119 per EVT^1^). We were able to feed one-third of our EVT-treated patients with liquid enteral nutrition. After the second week of EVT, only 20% of the patients needed tube or parenteral feeding. However, there is no cost advantage for stent therapy once a second stent is required, such as in cases of stent migration or inadequate leak sealing. Those cases are reported to occur in 30–50% of all stent therapy cases [1, 6–10, 13].

## Conclusion

Diagnosis of gastric leaks after bariatric surgery is hindered mainly because of excess visceral fat delays. We have shown that only early detection of GL (within 2 days after bariatric surgery) led to full healing after early revision surgery, suturing, and percutaneous drainage. For patients showing signs of GL beyond 2 days of primary surgery, revisional surgery was insufficient in all cases due to local peritonitis. For these patients, EVT resulted in a 90% healing rate (mean therapy time 17 days), normal weight loss, and no complications. EVT as standalone therapy in late leaks with good compartmentalization was as successful in 4 of 4 patients.

## Supplementary Information

Below is the link to the electronic supplementary material.

Supplementary file1 (PDF 365 kb)

Supplementary file2 (DOCX 25 kb)

## Footnotes

1 Prices are ascertained by research in internet.
