# Supplementary material for: Endoscopic vacuum therapy in salvage and standalone treatment of gastric leaks after bariatric surgery
Source: Langenbecks Arch Surg. 2021 Nov 17;407(3):1039–46. doi: 10.1007/s00423-021-02365-9 (PMC9151560; doi:10.1007/s00423-021-02365-9)
Supplement: Supplementary file 1 — Supplementary file1 (PDF 365 kb) [file 423_2021_2365_MOESM1_ESM.pdf]

MEDIZINISCHE FAKULTÄT
DER CHRISTIAN-ALBRECHTS-UNIVERSITÄT ZU KIEL

ETHIK-KOMMISSION

A.D. 1665

Universitäts-Kinderklinik · Schwanenweg 20 · 24105 Kiel

Postadresse:
Arnold-Heller-Straße 3 / Haus 9
D-24105 Kiel

Telefon 04 31 / 597-18 09
Telefax 04 31 / 597-53 33
ethikkomm@email.uni-kiel.de

Dr. med. Markus Ahrens
Klinik für Allgemeine, Visceral-, Thorax-,
Transplantations- und Kinderchirurgie
UKSH, Campus Kiel
Arnold-Heller-Str. 3, Haus 18
24105 Kiel

EINGEGANGEN
05. März 2013
Sekretariat: Prof. Dr. med. Thomas Becker

Datum: 25.02.2013

OA Ahrens!

AZ: D 427/13 (bitte stets angeben)

Studienplan: **Lebensqualitäts- und klinische Entwicklung nach konservativer und operativer Adipositastherapie**
Studienprotokoll, Fragebogen, Patienteninformation und Einwilligungserklärung

Datum des Antrages: **Eingang 19.02.2013**

Sehr geehrter Herr Kollege Ahrens,

vielen Dank für Ihren obengenannten Antrag zur Beratung gemäß § 15 Berufsordnung (BO) der Ärztekammer Schleswig-Holstein.

Nach Durchsicht der Unterlagen durch die Geschäftsstelle und durch mich als Vorsitzenden der Ethik-Kommission bestehen gegen die Durchführung der Studie keine berufsethischen und berufsrechtlichen Bedenken.

Es wird darauf hingewiesen, dass künftige Änderungen des Antrages der Ethik-Kommission anzuzeigen sind und gegebenenfalls eine erneute Beratung erforderlich machen.

Nach Abschluss der Studie erbittet die Kommission einen kurzen Bericht mit einem Hinweis, ob im Laufe der Studie ethische oder juristische Probleme aufgetreten sind.

Mit freundlichen kollegialen Grüßen

Prof. Dr. med. H. M. Mehdorn
Vorsitzender der Ethik-Kommission

Dr. med. Christine Glinicke
Geschäftsführung der Ethik-Kommission
