# Supplementary material for: Endoscopic vacuum therapy in salvage and standalone treatment of gastric leaks after bariatric surgery
Source: Langenbecks Arch Surg. 2021 Nov 17;407(3):1039–46. doi: 10.1007/s00423-021-02365-9 (PMC9151560; doi:10.1007/s00423-021-02365-9)
Supplement: Supplementary file 2 — Supplementary file2 (DOCX 25 kb) [file 423_2021_2365_MOESM2_ESM.docx]

Supplementary Table: Overview of 20 gastric leak patients treated with endovascular vacuum therapy (EVT).

| Patient | Operation (year) | BMI (kg/m^2^) | Type of operation | Gastric leak detection | Leak localization | Leak size* | EVT time (d) | Hospital stay after EVT (d) | Re-operation | ICU during EVT | Complications during follow-up | |
| --- | --- | --- | --- | --- | --- | --- | --- | --- | --- | --- | --- | --- |
|  |  |  |  |  |  |  |  |  |  |  | 3 months | 12 months |
| MB | 2013 | 60 | SG | Early | Distal | Micro | 3 | 7 | 1 | No | None** | None |
| CG | 2012 | 57 | SG | Early | Distal | Micro | 5 | 9 | 1 | No | None | None |
| NS | 2013 | 42 | SG | Early | Proximal | Macro | 28 | 3 | 1 | No | None | None |
| PA | 2012 | 64 | SG | Early | Proximal | Macro | 5 | 14 | 2 | No | None | None |
| SI | 2012 | 48 | SG | Early | Proximal | Macro | 33 | 22* | 1 | Yes | None | None |
| HP | 2011 | 61 | SG | Early | Proximal | Macro | 10 | 12 | 2 | No | None | None |
| GE | 2011 | 41 | RYGB | Late | Proximal | Macro | 12 | 10 | 0 | No | None | None |
| SK | 2011 | 50 | SG | Late | Proximal | Micro | 7 | 21** | 2 | Yes | None | None |
| KO | 2011 | 50 | SG | Late | Proximal | Micro | 7 | 14 | 1 | No | None | None |
| CGa | 2015 | 45 | SG | Early | Proximal | Micro | 55 | 30*** | 2 | Yes | None | None |
| DP | 2016 | 52 | RYGB | Early | Proximal | Macro | 21 | 44**** | 1 | No | None | None |
| JH | 2016 | 56 | SG | Late | Proximal | Micro | 36 | 7 | 1 | No | None | None |
| MG | 2016 | 43 | RYGB | Early | Proximal | Macro | 20 | 7 | 1 | No | None | None |
| AB | 2016 | 47 | RYGB | Late | Proximal | Micro | 12 | 4 | 0 | No | None | None |
| PG | 2017 | 48 | RYGB | Early | Proximal | Micro | 19 | 6 | 0 | No | None | None |
| KL | 2017 | 51 | RYGB | Early | Proximal | Micro | 38 | 10 | 1 | No | None | None |
| SR | 2017 | 53 | SG | Late | Proximal | Micro | 19 | 2 | 0 | No | None | None |
| SZ | 2017 | 57 | MGB | Late | Proximal | Micro | 4 | 4 | 0 | No | None | None |
| PS | 2013 | 54 | SG | Late | Proximal | Macro | Gastrectomy | — | 4 | — | None | None |
| DM | 2013 | 60 | SG | Late | Proximal | Macro | Died after 4 weeks | — | 2 | — | — | — |

The therapy time of micro leaks was 3-7 days. None of the patients had any kind of specific or EVT-associated complications during follow up.

* Leak size: micro = only indirect leak signs proofed by operation, radiology or gastroscopy; macro = direct proof of leak.

** None = no stenosis, no dysphagia, no de novo GERD, no abnormal postoperatively reduced food intake.

*** Patient with multiple comorbidities, e.g. terminal kidney insufficiency and diabetes.

**** Patient suffered a pancreatic fistula caused by a perisplenic abscess.

RYGB, Roux-en-Y gastric bypass; MGB, mini-gastric bypass; SG, sleeve gastrectomy.
